# Suspected microdeletion syndromes and molecular cytogenetic techniques: an experience with 330 cases

**DOI:** 10.1186/1755-8166-7-S1-O7

**Published:** 2014-01-21

**Authors:** Ashutosh Halder, Manish Jain, Isha Chaudhary

**Affiliations:** 1 Department of Reproductive Biology, AIIMS, New Delhi 110029, India

## Background

Microdeletion syndromes are characterized by small (< 5Mb) chromosomal deletion in which one or more genes are involved. They are frequently associated with multiple congenital anomalies. The phenotype is the result of haploin sufficiency of genes in the critical interval. Fluorescent In-Situ Hybridization (FISH), Multiplex Ligation-dependent Probe Amplification (MLPA), Quantitative Fluorescent Polymerase Chain Reaction (QFPCR) and Array (microarray) Comparative Genomic Hybridization (aCGH) techniques are commonly used for precise genetic diagnosis of microdeletion syndromes.

## Methods

This study comprised of 330 cases of suspected microdeletion syndromes. There were 184 cases of 22q11.2 microdeletion, 52 cases of William, 47 cases of Prader Willi/Angelman, 18 cases of Miller Dieker, 14 cases of Retinoblastoma (bilateral infantile), 5 cases of Trichorhinophalangeal (TRP) and 10 cases of other microdeletion syndromes. FISH was carried out on all 330 clinically suspected microdeletion syndrome cases using non-commercial FISH probes. Subsequently, we have performed aCGH in 100 cases (77 cases of 22q11.2; 9 cases of William Syndrome, 8 cases of Prader Willi Syndrome, 4 cases of Miller Dieker Syndrome and 2 cases of other microdeletion syndromes) including one monozygotic twin pairs with discordant phenotype. Another 50 cases, including 22q11.2 microdeletion, aCGH experiment and analysis are in progress.

## Results

FISH was confirmatory in 28 cases only (8.48%; 19 cases of 22q11.2 microdeletion, 5 cases of Prader Willi, 3 cases of William and 1 case of TRP syndrome). There were 8 cases with mosaicism and 20 cases with pure deletion. Microarray was picked up copy number variation (CNV) with or without copy neutral loss of heterozygosity (LOH) in approximately 70% of cases, mostly involving several chromosome loci. However, aCGH was failed to pick up mosaic cases (with even 45% deleted cell lines). Clinically suspected specific locus CNV was detectable in approximately 24% cases only by aCGH. Variation in deletion sizes and or break point differences (with genes involvement variations) as well as other CNVs with or without LOH was evident.

## Conclusions

We conclude that FISH in this format should not be the method of choice for clinically suspected microdeletion syndromes as cost, labor & time versus benefit is unjust. Microarray seems better technique, in clinically doubtful cases. However, microarray is likely going to miss mosaic cases, if deleted cell lines concentration is less than 50%. It seems time has come to follow strict clinical criteria for FISH testing or preferably to follow better methods viz., DNA microarray (array comparative genomic hybridization). We think that whole genome screening should be adopted as first line of investigation and FISH may be used for detecting mosaicism, screening family members and prenatal diagnosis. Furthermore, microdeletion syndrome best fitted with genomic disorder as several chromosomal loci are involved in CNV with or without LOH and alteration in deletion size or breakpoint. Our study has not found identical deletion profile in any cases, thus explaining reason for phenotypic variability between deletion positive cases.

